# Efficacy of hyperthermic intrathoracic chemotherapy for initially diagnosed lung cancer with symptomatic malignant pleural effusion

**DOI:** 10.1038/s41598-023-39211-5

**Published:** 2023-07-26

**Authors:** Zihui Li, Jie Deng, Fei Yan, Li Liu, Yanling Ma, Jianhai Sun

**Affiliations:** Oncology Department, The Third People’s Hospital of Hubei Province, Affiliated Hospital of Jianghan University, 26# Zhongshan Avenue, Qiaokou District, Wuhan, 430033 Hubei Province China

**Keywords:** Cancer, Medical research, Risk factors

## Abstract

Initially diagnosed malignant pleural effusion (MPE) has different systematic treatments, and defining the best drainage regimen according to the responsiveness of MPE to different systematic treatments is important. This study compared the efficacy of hyperthermic intrathoracic chemotherapy (HITHOC) and pleural catheter drainage (IPCD) for initially diagnosed lung cancer with symptomatic MPE. We retrospectively reviewed the medical records of initially diagnosed lung cancer patients with symptomatic MPE between January 2018 and May 2022. The patients were treated with IPCD or HITHOC for local control of MPE after diagnosis. Systematic regimens were conducted during 1 month according to guidelines after local treatment. Intrathoracic MPE progression-free survival (iPFS) and overall survival (OS) were calculated, Univariate and multivariable Cox-regression were used to identify factors associated with iPFS and OS. A total of 33 patients were evaluated; 10 (30.3%) patients received IPCD, and 23 (69.7%) patients received HITHOC. No difference in the MPE control rate at 1 month was found between the IPCD group (90%) and HITHOC group (95.7%). However, this control rate was significantly higher in the HITHOC group (69.6%) than in the IPCD group (30%) at 3 months (P = 0.035). Multivariate analysis showed that receiving tyrosine kinase inhibitors (TKIs) or chemotherapy was a significant protective factor for iPFS (HR = 0.376, 95% CI 0.214–0.659, P = 0.007) and OS (HR = 0.321, 95% CI 0.174–0.594, P < 0.001). According to subgroup analysis, among patients treated with TKIs, those who received HITHOC had longer iPFS and OS than those who received IPCD (P = 0.011 and P = 0.002, respectively), but this difference was not found in the palliative care subgroup. Moreover, no patients treated with chemotherapy showed reaccumulation of MPE. Systematic TKIs or chemotherapy prolonged iPFS and OS for those initially diagnosed with lung cancer with symptomatic MPE. HITHOC prolonged iPFS and OS for those treated with systematic TKIs.

## Introduction

MPE is seen in 15–24% of patients with non-small cell lung carcinoma (NSCLC) at diagnosis, and this proportion increases with disease progression^1^. Classified as stage IV, lung cancer patients with massive MPE can display dyspnea, chest discomfort, and cough, and their median survival is four months, which is shorter than that of patients with other types of metastasis^2,3^. Local treatments for MPE include pleurodesis, placement of an indwelling pleural catheter, and pleuroscopy with chemical pleurodesis^4–6^. In addition to local treatment, systematic treatments, such as TKIs, chemotherapy, and anti-vascular endothelial growth factor (anti-VEGF), have shown efficacy in MPE. Although the landscape of systematic management for lung cancer has changed significantly over the last decade, local treatment recommended for MPE continues to lag^7^. NCCN guidelines refer to pleurodesis or ambulatory small catheter drainage for local therapy in suitable patients. Nevertheless, only 60% of MPE patients achieve temporary relief, with most experiencing recurrence in 3 months. Since initially diagnosed MPE involves different systematic treatments, defining the best drainage regimen according to the responsiveness of MPE to different systemic treatments is important.

HITHOC is a local treatment for MPE. Through a specially designed pump, HITHOC infuses constant-temperature (43 °C) drugs for circulation in the thoracic cavity. Thus, HITHOC suppresses tumor growth not only by the physical mechanism of flushing out the chest but also by the synergistic biological effect of hyperthermia and antineoplastic drugs^8^. HITHOC has been proven to be effective in various kinds of hydrothorax and ascites with metastasis. In lung cancer, HITHOC combined with video-assisted thoracic surgery (VATS) was proposed as a radical treatment for individuals with lung cancer-associated MPE but no distant metastasis^6^. When neglect of systemic treatment systemic treatment, exclusive HITHOC of endostatin and nedaplatin prolonged the time to MPE progression, and the efficacy and safety are reported to be comparable to those of talc pleurodesis^9,10^. However, the efficacy of HITHOC in internal medicine for initially diagnosed lung cancer with symptomatic MPE has not been reported.

In this study, we retrospectively reviewed the local and systematic treatment records of 33 patients with initially diagnosed MPE in advanced lung cancer. We aimed to assess the efficacy of HITHOC in individuals with initially diagnosed symptomatic MPE in lung cancer to provide an additional reference for clinical treatment strategies.

## Materials and methods

### Study population

This was a single-center retrospective study of all consecutive patients with newly diagnosed metastatic lung cancer with MPE between January 2018 and May 2022 at the Third People’s Hospital of Hubei Province. This study was approved by the local ethics committee in accordance with the ethical principles of the Declaration of Helsinki. Informed consent was obtained from all patients before treatment.

Thirty-three patients were enrolled in this study. The inclusion criteria were as follows: (1) newly diagnosed lung cancer with symptomatic MPE without systematic treatment; (2) pathologically proven lung cancer, pleural fluid positive for tumors or showing elements that the effusion is highly related to the tumor, including the fluid being bloody or tumor markers in MPE significantly increased and synchronously increased with those in blood; (3) Eastern Cooperative Oncology Group Performance Status (ECOG PS) score ranging from 0 to 3; and (4) distant metastasis ineligible for surgery. Patients were excluded if they met one of the following criteria: (1) contraindications for HITHOC, such as severe heart condition or abnormal coagulation function; (2) ECOG PS score of 4; or (3) incomplete follow-up information.

### Treatment procedure

In all patients, with an infusion catheter was inserted into the pleural cavity under B-ultrasound guidance to alleviate dyspnea immediately. The hydrothorax sample was sent for biochemical and cytological examination. Genetic testing was recommended for the best systematic regimens if the pleural fluid was positive for tumor cells. Patients in the IPCD group underwent pleural fluid drainage every other day or according to their symptoms. Patients in the HITHOC group received an outflow catheter for HITHOC.

The detailed operating process was performed as previously described^9,11^. Briefly, one session of HITHOC therapy consisted of three sessions that operated on the first, third, and fifth days after diagnosis. Every session of HITHOC consisted of four cycles of circulation, up to 15 min depending on the flow rate and the patient’s comfort. The hydrothorax was removed before HITHOC treatment. When the solution of 0.9% sodium chloride was heated to 42.5 °C–43 °C, the infusion and outflow catheter were connected to the hyperthermic perfusion treatment system (RHL-2000A, Jilin, China) for circulation. The flow rate was set to 40–60 mL/min. Lobaplatin (50 mg for the second cycle in one or two sessions) was also injected into the chest cavity for circulation. Based on the patients’ ECOG PS score and desire, Endostar (60 mg) was injected into the chest the day after every session. The temperature was measured by temperature-monitoring probes to ensure the targeted 42.5 °C–43 °C. Four vital signs were assessed using a multiparameter patient monitor.

Hydrothorax and blood were used for biochemical examination before and after thoracentesis. If symptoms were not completely relieved within 30 days, a second placement of an indwelling pleural catheter or HITHOC was suggested.

### Systematic treatment and follow-up

After completion of the first HITHOC, patients received EGFR-targeted TKIs, systematic chemotherapy, or palliative care according to the results of the genetic test. Patients in the TKI group received TKIs and were followed up at 1, 3, 6, and 12 months in the first year and every 3 months in the second year. Patients in the chemotherapy group had no EGFR mutation and received chemotherapy with pemetrexed, paclitaxel, and etoposide combined with platinum according to their pathology pattern. Patients in the palliative care group received no systematic anticancer treatment. At least every 2–3 weeks, B-ultrasound was performed to assess the therapeutic efficacy of local treatment in the first 3 months after treatment. All subsequent treatment options for progression were determined according to the guidelines.

### Efficacy assessments

**Figure 1:**
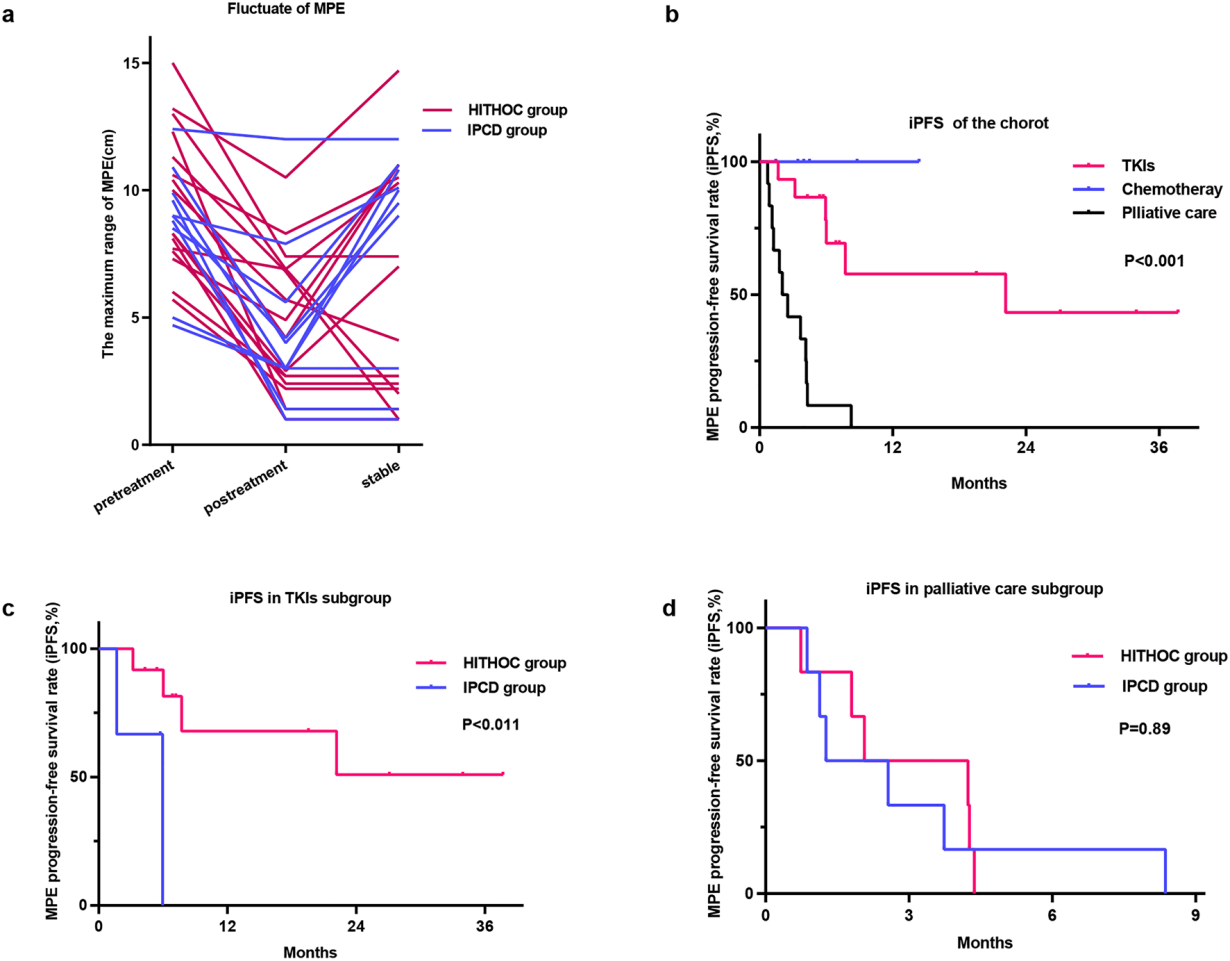
Fluctuations in MPE and intrathoracic MPE progression-free survival in patients with different treatments. (**a**) Fluctuation of the maximum MPE detected by B-ultrasound before and after first-line treatment. (**b**) Intrathoracic MPE progression-free survival in patients treated with TKIs, chemotherapy, or palliative care treatment. ((**c**) and (**d**)) Impact of local treatment on MPE progression-free survival in patients treated with TKIs (**c**) or palliative care (**d**) in subgroup analysis. *MPE* malignant pleural effusions, *TKI* tyrosine kinase inhibitor, *HITHOC* hyperthermic intrathoracic chemotherapy, *IPCD* indwelling pleural catheter drainage.

The stability of MPE is defined as reaccumulated MPE as much as before the first local treatment or no trend of reaccumulation for at least 1 month after all sessions of local treatment (Fig. 1a). We also assessed the MPE control rate at 1 and 3 months, intrathoracic MPE progression-free survival (iPFS) and overall survival. Recent objective responses regarding the MPE control rate for 1 and 3 months were determined according to modified WHO criteria for efficacy assessment in malignant tumors: complete remission (CR) was considered when the accumulated fluid had disappeared and was sustained for 4 weeks; partial remission (PR) was considered if pleural effusions were reduced by 50% and remained for 4 weeks; stable disease (SD) was considered if the pleural effusions were not reduced; progressive disease (PD) was considered when the pleural effusions increased. MPE control was calculated by taking the sum of CR + PR. Long-term responses of iPFS were defined as the interval from the indwelling pleural catheter to reaccumulation of MPE, death from any cause, or the last follow-up visit. OS was defined as the period from the indwelling pleural catheter until the death of the patient from any cause.

### Statistical analysis

Statistical analysis was performed using SPSS 27.0 and GraphPad Prism 7. Continuous variables are expressed as medians (range); frequencies and percentages are used for categorical data. Differences in clinical data were evaluated using the Mann‒Whitney U test, χ^2^ test, or Fisher’s exact probability test. iPFS and OS were estimated using Kaplan‒Meier curves, and the log-rank test was used for comparisons. Prognostic factors associated with iPFS and OS were investigated by a Cox regression model. Significant variables (P values < 0.10) identified by the univariate analyses were included in a multivariate Cox proportional hazards model. A two-tailed P value < 0.05 was considered statistically significant.

## Results

### Patient characteristics

The patients’ baseline characteristics are summarized in Table 1, as stratified according to local treatment of IPCD and HITHOC. Ten (30.3%) patients received IPCD, and 23 (69.7%) patients received HITHOC. The maximum depth of MPE at diagnosis was 9 cm (4.7–12.4) and 8.2 cm (5.7–15.0) in the IPCD and HITHOC groups, respectively. The HITHOC group had more patients with adenocarcinoma than the IPCD group (P = 0.023). Other parameters of patient characteristics as well as systematic anticancer treatment regimens were comparable between the two groups.

**Table 1:** Baseline characteristics and short-term efficacy.

Characteristics	IPCD group (n = 10)	HITHOC group (n = 23)	p-value
Age, years	79 (56–89)	69 (53–84)	0.11
Gender			
Male	1 (10)	9 (39.1)	0.094
Female	9 (90)	14 (60.9)	
Maximum depth of MPE at diagnosis, cm	9 (4.7–12.4)	8.2 (5.7–15.0)	0.11
Pathology			
Lung adenocarcinoma	6 (60)	22 (95.7)	0.023
Lung squamous cell carcinoma	2 (20)	1 (4.3)	
SCLC	2 (20)	0 (0)	
Driver gene			
EGFR postive	2 (20)	10 (43.5)	0.070
EGFR negative	3 (30)	10 (43.5)	
Unknown	5 (50)	3 (13)	
ECOG PS scores at diagnosis			
1	2 (20)	9 (39.1)	0.545
2	5 (50)	8 (34.8)	
3	3 (30)	6 (26.1)	
Systemic treatment regimens			
Tyrosine kinase inhibitors	3 (30)	12 (52.2)	0.175
Chemotherapy	1 (10)	5 (21.7)	
Palliative care	6 (60)	6 (26.1)	
Short-term efficacy			
MPE control rate in 1 month			
No	1 (10)	1 (4.3)	0.532
Yes	9 (90)	22 (95.7)	
MPE control rate in 3 months			
No	3 (30)	16 (69.6)	0.035
Yes	7 (70)	7 (30.4)	

Data are presented as No. (%), unless otherwise indicated.

*HITHOC* Hyperthermic intrathoracic chemotherapy, *MPE* malignant pleural effusion, *SCLC* small cell lung cancer, *EGFR* epidermal growth factor receptor, *ECOG PS scores* Eastern Cooperative Oncology Group performance status scores, *TKI* tyrosine kinase inhibitor.

### Short-term efficacy

All patients showed alleviation of their symptoms after the first IPCD or HITHOC. There was no difference in the MPE control rate between the IPCD (90%) and HITHOC (95.7%) groups at 1 month (P = 0.532, Table 1). However, the MPE control rate in the IPCD group decreased to 30% in the third month, which was significantly (P = 0.035) lower than that in the HITHOC group (69.6%). The fluctuation of MPE is shown in Fig. 1a.

### Intrathoracic MPE progression-free survival and subgroup analysis

The median time to MPE recurrence in the IPCD group was 2.5 months, which was shorter (P < 0.005) than that in the HITHOC group (22.4 months). Although receiving HITHOC treatment was an independent protective factor for iPFS in univariate analysis (HR = 0.218, 95% CI = 0.082–0.584, P = 0.002), only receiving systematic chemotherapy or TKI (HR = 0.376, 95% CI = 0.214–0.659, P = 0.007) was an independent protective factor for MPE reaccumulation (Table 2). The Kaplan‒Meier plot showed that the iPFS rate at 1 and 2 years was the highest in the chemotherapy group, followed by the TKI group and palliative care group (P < 0.001, Fig. 1b). Subgroup analysis stratified by different systematic treatments showed that the HITHOC group had significantly longer iPFS than the IPCD group in the TKI subgroup (P = 0.011, Fig. 1c), but no difference was found in the palliative care subgroup (P = 0.89, Fig. 1d). Notably, no patients treated with chemotherapy showed reaccumulation of MPE.

**Table 2:** Univariate and multivariate analysis of prognostic factors influencing iPFS in cox proportional hazards model.

Variable	Univariate analysis		Multivariate analysis	
	HR (95% CI)	p-value	HR (95% CI)	p-value
Sex	3.118 (0.872–11.143)	0.080		
Age	1.056 (1.002–1.113)	0.039		
Local treatment for MPE	0.273 (0.103–0.724)	0.009		
Maximum depth of MPE at diagnosis	1.062 (0.914–1.234)	0.430		
Pathology	1.848 (0.849–4.022)	0.121		
Driver gene	2.117 (1.076–4.166)	0.030		
ECOG PS scores	2.093 (1.129–3.879)	0.019		
Systemic treatment regimen	0.321 (0.174–0.594)	< 0.001	0.321 (0.174–0.594)	< 0.001

*MPE* malignant pleural effusion, *ECOG PS scores* Eastern Cooperative Oncology Group performance status scores.

### Overall survival and subgroup analysis

**Figure 2:**
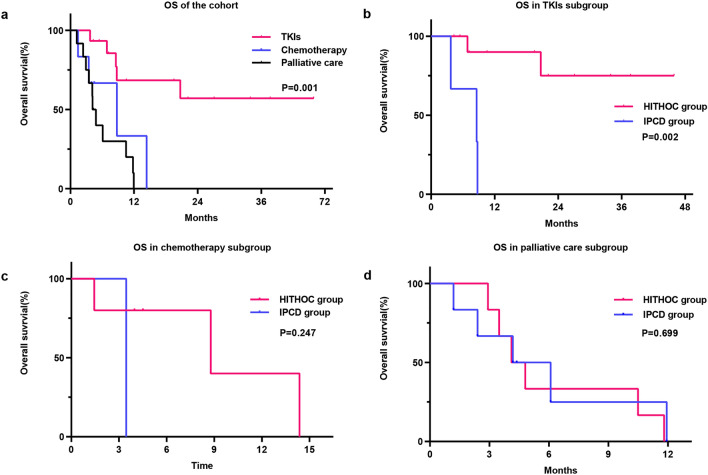
Overall survival in patients with different treatments. (**a**) Overall survival in patients treated with TKIs, chemotherapy, or palliative care treatment. (**b–d**) Impact of local treatment on overall survival in patients treated with TKIs (**b**), chemotherapy (**c**), or palliative care treatment (**d**) in subgroup analysis. *MPE* malignant pleural effusions, *TKI* tyrosine kinase inhibitor, *HITHOC* hyperthermic intrathoracic chemotherapy, *IPCD* indwelling pleural catheter drainage.

According to univariate analysis, older age, IPCD treatment, no driver genes, high ECOG PS score, and palliative care treatment were associated with an increased risk of mortality. However, in multivariate analysis, only receiving systematic chemotherapy or TKI (HR = 0.321, 95% CI = 0.174–0.594, P < 0.001) was an independent protective factor for OS (Table 3, Fig. 2a P = 0.001). Subgroup analysis showed that patients in the HITHOC group survived longer than those in the IPCD group among patients treated with TKIs (P = 0.002, Fig. 2b). However, this survival advantage was not found in the palliative care subgroup (P = 0.699, Fig. 2d). No significant difference in OS was observed between patients treated with HITHOC and IPCD in the chemotherapy subgroup, but there was a trend that patients treated with HITHOC survived longer than those treated with IPCD (P = 0.247, Fig. 2c).

**Table 3:** Univariate and multivariate analysis of prognostic factors influencing OS in cox proportional hazards model.

Variable	Univariate analysis		Multivariate analysis	
	HR (95% CI)	p-value	HR (95% CI)	p-value
Sex	2.058 (0.682–6.212)	0.200		
Age	1.049 (0.999–1.102)	0.057		
Local treatment for MPE	0.218 (0.082–0.584)	0.002		
Maximum depth of MPE at diagnosis	0.971 (0.834–1.131)	0.705		
Pathology	2.365 (1.165–4.804)	0.017		
Driver gene	3.081 (1.632–5.817)	0.001		
ECOG PS scores	2.173 (1.161–4.067)	0.015		
Systemic treatment regimen	0.376 (0.214–0.659)	0.001	0.376 (0.214–0.659)	0.001

*MPE* malignant pleural effusion, *ECOG PS scores* Eastern Cooperative Oncology Group performance status scores.

## Discussion

This is the first study to evaluate the efficacy of HITHOC for initially diagnosed lung cancer with MPE. Our findings showed that systematic TKIs or chemotherapy prolonged iPFS and OS for those initially diagnosed with lung cancer with symptomatic MPE. Although HITHOC was not an independent factor for MPE iPFS and OS in multivariate analysis, subgroup analysis showed that patients treated with HITHOC had longer iPFS and OS than those treated with IPCD among individuals receiving systematic management of TKIs.

The most appropriate approach for MPE is determined by the procedure’s risks, postdrainage rate of fluid reaccumulation, extent of lung entrapment, and patient's expected survival. IPCD and its developed intrathoracic local treatment have been acknowledged as the worldwide first-line management options for MPE due to the considerable rate of pleurodesis and the ease of use for further invasive pleural procedures^12,13^. HITHOC combined with VATS has been suggested for highly selected initially diagnosed NSCLC with pleural effusion but with no distant metastasis for radical purposes. However, some patients cannot tolerate VATS anesthesia and have surgery risks^14^. The HITHOC in our study avoided the surgery of VATS with insertion of an infusion and outflow catheter under B-ultrasound guidance to keep the drug solution circulating in the thoracic cavity. Consistent with a previous study, the response rate in the HITHOC group at one month was 95.7%, which was comparable to that in HITHOC combined with VATS^6^. The HITHOC group also achieved a higher 3-month sustainable MPE progression-free rate than the IPCD group (69% vs*.* 30%). Moreover, the median iPFS in the HITHOC group was 22.4 months, markedly longer than that in previous studies (ranging from 3.6 to 4.2 months)^9,10^^.^

Despite the reliability of HITHOC in MPE control, only receiving TKIs or chemotherapy was a significant protective factor for iPFS and OS in multivariate analysis. In recent decades, the PFS and OS of patients with MPE have generally increased since TKI and chemotherapy were applied in the clinic. Previous studies have focused on local intrathoracic treatment and ignored the value of systematic treatment for long-term MPE alleviation. Uncontrollable MPE and repeated drainage may prolong MPE-related symptoms, which leads to loss of the opportunity for further systematic treatment. Defining the best local treatment according to genetic testing and systematic treatment is important.

Patients with MPE treated with TKIs often show an initial reduction, and almost 70% of them will experience recurrence requiring palliative intervention^15–17^. MPE often responds worse to EGFR TKIs than solid tumors^18,19^. One study reported significant accumulation of both erlotinib and OSI-420 in MPE with repeated dosing; thus, concentrations of TKIs in MPE do not appear to be responsible for unsatisfactory regression^20^. The partial resistance of MPE to TKI treatment suggests intrinsic factors affecting the therapeutic efficacy, such as activation of VEGF signaling, enrichment of putative cancer stem cells in MPE ^21^, and primary resistance by cooccurring genetic alterations such as Kras^22^. EGFR mutations are related to a higher risk of MPE recurrence after initial thoracentesis at 30 and 100 days than in the absence of EGFR mutation^11^. Mechanistically, more radical local intrathoracic therapy can be an effective option for better prognosis for those with EGFR mutations. In our study, patients treated with TKIs combined with HITHOC achieved significantly longer iPFS and OS than those treated with TKIs combined with IPCD.

Despite the small sample size, all patients treated with systematic chemotherapy and HITHOC in our study showed no reaccumulation of MPE. An early study researched the efficacy of intrapleural chemotherapy with cisplatin and cytarabine. Twenty-eight patients (80.0%) maintained complete control of MPE until death or the last follow-up^23^. Despite the serious adverse effects that can currently be well managed, the efficacy in Kim’s study suggests that chemotherapy may be essential for effective control of MPE, whether with or without EGFR mutation. A prospective trial showed that carboplatin and paclitaxel with bevacizumab have a controlled rate of 91.3% during chemotherapy for MPE, and the PFS and OS were 7.1 months and 11.7 months, respectively^24^. Another study showed that carboplatin and pemetrexed with bevacizumab are effective for treating MPE, with a controlled rate of 92.9% at 8 weeks; PFS and OS were 8.2 months and 18.6 months, respectively^25^. Considering the relatively sustainable efficacy for MPE control for those treated with systematic chemotherapy and unsatisfactory regression in EGFR mutations, it is important to explore the radical cycles of chemotherapy for MPE NSCLC patients with EGFR mutation.

This was a retrospective study performed in a single institution. The sample size was relatively small, and two SCLC patients were included in our study, which pathology conferring relatively poor long-term prognosis. Moreover, differences between drug use in HITHOC were not compared in palliative care subgroups. However, our study demonstrated that HITHOC can prolong the iPFS and OS of initially diagnosed MPE patients treated with TKIs, providing an additional reference for clinical treatment strategies. More safe and effective treatment regimens for initially diagnosed MPE according to different pathologies for lung cancer remain to be explored.

## Data Availability

Data that support the fundings of this study are available from the corresponding author upon reasonable request.

## References

[1] Porcel JM (2015). Clinical features and survival of lung cancer patients with pleural effusions. Respirology.

[2] Mishra EK (2021). Breathlessness predicts survival in patients with malignant pleural effusions: Meta-analysis of individual patient data from five randomized controlled trials. Chest.

[3] Quek JC, Tan QL, Allen JC, Anantham D (2020). Malignant pleural effusion survival prognostication in an Asian population. Respirology.

[4] Asciak R, Rahman NM (2018). Malignant pleural effusion: From diagnostics to therapeutics. Clin. Chest Med..

[5] Bhatnagar R (2018). Outpatient talc administration by indwelling pleural catheter for malignant effusion. N. Engl. J. Med..

[6] Wang X (2022). The efficacy and safety of intrapleural hyperthermic perfusion in patients with malignant pleural effusion undergoing video-assisted thoracic surgery: A single-arm clinical trial. J. Thorac. Dis..

[7] Feller-Kopman DJ (2018). Management of malignant pleural effusions. An official ATS/STS/STR clinical practice guideline. Am. J. Respir. Crit. Care Med..

[8] Li Z, Deng J, Sun J, Ma Y (2020). Hyperthermia targeting the tumor microenvironment facilitates immune checkpoint inhibitors. Front. Immunol..

[9] Chen L, Zhu X, Li D, Cai X (2020). Effect of thoracic hyperthermic perfusion with recombinant human endostatin plus nedaplatin in treating pleural effusion in patients with advanced non-small cell lung cancer. J. BUON.

[10] Kleontas A (2019). Clinical factors affecting the survival of patients diagnosed with non-small cell lung cancer and metastatic malignant pleural effusion, treated with hyperthermic intrathoracic chemotherapy or chemical talc pleurodesis: A monocentric, prospective, randomized trial. J. Thorac. Dis..

[11] Schwalk AJ (2021). Risk factors for and time to recurrence of symptomatic malignant pleural effusion in patients with metastatic non-small cell lung cancer with EGFR or ALK mutations. Chest.

[12] Muruganandan S (2018). Aggressive versus symptom-guided drainage of malignant pleural effusion via indwelling pleural catheters (AMPLE-2): An open-label randomised trial. Lancet Respir. Med..

[13] Thomas R (2017). Effect of an indwelling pleural catheter vs talc pleurodesis on hospitalization days in patients with malignant pleural effusion: The AMPLE randomized clinical trial. JAMA.

[14] Mollberg NM (2012). Quality of life after radical pleurectomy decortication for malignant pleural mesothelioma. Ann. Thorac. Surg..

[15] Kashiwabara K, Fuji S, Tsumura S, Sakamoto K (2020). Prognosis of EGFR-mutant lung adenocarcinoma patients with malignant pleural effusion receiving first-line EGFR-TKI therapy without pleurodesis: A single-institute retrospective study. Anticancer Res..

[16] Lin JB (2017). Sequential treatment strategy for malignant pleural effusion in non-small cell lung cancer with the activated epithelial grow factor receptor mutation. J. Drug Target..

[17] Wang W, Jiang X, Zhang Y, Song Y, Song Z (2019). Intracavitary chemotherapy with epidermal growth factor receptor-tyrosine kinase inhibitor (EGFR-TKI) is not superior to TKI monotherapy in controlling malignant pleural effusion recurrence in EGFR-mutated lung cancer patients. J. Thorac. Dis..

[18] Kobayashi K (2020). Key prognostic factors for EGFR-mutated non-adenocarcinoma lung cancer patients in the Japanese Joint Committee of Lung Cancer Registry Database. Lung Cancer.

[19] Yang J (2018). EGFR mutation status in lung adenocarcinoma-associated malignant pleural effusion and efficacy of EGFR tyrosine kinase inhibitors. Cancer Res. Treat..

[20] Masago K (2011). Plasma and pleural fluid pharmacokinetics of erlotinib and its active metabolite OSI-420 in patients with non-small-cell lung cancer with pleural effusion. Clin. Lung Cancer.

[21] Basak SK (2009). The malignant pleural effusion as a model to investigate intratumoral heterogeneity in lung cancer. PLoS One.

[22] DeMaio A (2019). Yield of malignant pleural effusion for detection of oncogenic driver mutations in lung adenocarcinoma. J. Bronchol. Interv. Pulmonol..

[23] Kim KW (2004). Intrapleural chemotherapy with cisplatin and cytarabine in the management of malignant pleural effusion. Cancer Res. Treat..

[24] Kitamura K (2013). Bevacizumab plus chemotherapy for advanced non-squamous non-small-cell lung cancer with malignant pleural effusion. Cancer Chemother. Pharmacol..

[25] Usui K (2016). A phase II study of bevacizumab with carboplatin-pemetrexed in non-squamous non-small cell lung carcinoma patients with malignant pleural effusions: North East Japan Study Group Trial NEJ013A. Lung Cancer.

